# Deep Learning for Identification of Acute Illness and Facial Cues of Illness

**DOI:** 10.3389/fmed.2021.661309

**Published:** 2021-07-26

**Authors:** Castela Forte, Andrei Voinea, Malina Chichirau, Galiya Yeshmagambetova, Lea M. Albrecht, Chiara Erfurt, Liliane A. Freundt, Luisa Oliveira e Carmo, Robert H. Henning, Iwan C. C. van der Horst, Tina Sundelin, Marco A. Wiering, John Axelsson, Anne H. Epema

**Affiliations:** ^1^Department of Clinical Pharmacy and Pharmacology, University Medical Center Groningen, University of Groningen, Groningen, Netherlands; ^2^Department of Anesthesiology, University Medical Center Groningen, University of Groningen, Groningen, Netherlands; ^3^Bernoulli Institute for Mathematics, Computer Science and Artificial Intelligence, University of Groningen, Groningen, Netherlands; ^4^Department of Intensive Care Medicine, Maastricht University Medical Centre+, University Maastricht, Maastricht, Netherlands; ^5^Department of Psychology, Stress Research Institute, Stockholm University, Stockholm, Sweden; ^6^Department of Clinical Neuroscience, Karolinska Institutet, Stockholm, Sweden

**Keywords:** gestalt, deep learning, facial analysis, synthetic data, acute illness

## Abstract

**Background:** The inclusion of facial and bodily cues (clinical gestalt) in machine learning (ML) models improves the assessment of patients' health status, as shown in genetic syndromes and acute coronary syndrome. It is unknown if the inclusion of clinical gestalt improves ML-based classification of acutely ill patients. As in previous research in ML analysis of medical images, simulated or augmented data may be used to assess the usability of clinical gestalt.

**Objective:** To assess whether a deep learning algorithm trained on a dataset of simulated and augmented facial photographs reflecting acutely ill patients can distinguish between healthy and LPS-infused, acutely ill individuals.

**Methods:** Photographs from twenty-six volunteers whose facial features were manipulated to resemble a state of acute illness were used to extract features of illness and generate a synthetic dataset of acutely ill photographs, using a neural transfer convolutional neural network (NT-CNN) for data augmentation. Then, four distinct CNNs were trained on different parts of the facial photographs and concatenated into one final, stacked CNN which classified individuals as healthy or acutely ill. Finally, the stacked CNN was validated in an external dataset of volunteers injected with lipopolysaccharide (LPS).

**Results:** In the external validation set, the four individual feature models distinguished acutely ill patients with sensitivities ranging from 10.5% (95% CI, 1.3–33.1% for the skin model) to 89.4% (66.9–98.7%, for the nose model). Specificity ranged from 42.1% (20.3–66.5%) for the nose model and 94.7% (73.9–99.9%) for skin. The stacked model combining all four facial features achieved an area under the receiver characteristic operating curve (AUROC) of 0.67 (0.62–0.71) and distinguished acutely ill patients with a sensitivity of 100% (82.35–100.00%) and specificity of 42.11% (20.25–66.50%).

**Conclusion:** A deep learning algorithm trained on a synthetic, augmented dataset of facial photographs distinguished between healthy and simulated acutely ill individuals, demonstrating that synthetically generated data can be used to develop algorithms for health conditions in which large datasets are difficult to obtain. These results support the potential of facial feature analysis algorithms to support the diagnosis of acute illness.

## Introduction

It is estimated that patients with sepsis alone account for as much as 6% of all hospital admissions and that while case-fatality rates are declining, the incidence of sepsis keeps increasing (1, 2). Early recognition of acute illness is critical for timely initiation of treatment (1). However, patients admitted to the emergency department (ED) or intensive care unit (ICU) with critical conditions such as sepsis often present with heterogeneous signs and symptoms, making detection and diagnosis challenging (3). Numerous risk scores based on laboratory variables and vital signs have been developed in an attempt to tackle this, but these achieved variable performance or were inferior to clinicians' informed judgment, also known as the clinical gestalt (4–7).

The clinical gestalt theory states that healthcare practitioners can actively organize clinical perceptions into coherent constructs or heuristics to reduce decision complexity, for example, by analyzing patients' facial and bodily cues, to estimate their functional status (8, 9). The value of the clinical gestalt as a diagnostic tool has been studied in different health conditions (10–13). In acute coronary syndrome, heart failure, pneumonia, and COVID-19, the clinical gestalt registered by doctors was comparable to clinical scores in “ruling in” or “ruling out” patients with certain symptoms presenting to the ED (10–14). For sepsis, the Third International Consensus Definitions for Sepsis and Septic Shock (Sepsis-3) advocates clinicians should, in addition to systemic inflammatory response syndrome (SIRS) criteria, use clinical gestalt in screening, treating and risk-stratifying patients with infection (15).

The clinical gestalt is also increasingly used as the basis for building deep learning models, with facial pictures being used to identify different genetic syndromes (16), as well as to detect coronary artery disease in an emergency setting (17). However, despite a growing number of studies reporting good results of deep learning models trained with a variety of clinical measurements to predict or detect early sepsis, no model has yet included clinical gestalt or facial feature analysis (18, 19). One major challenge to the development of a well-performing deep learning algorithm for facial analysis is the datasets' size and quality of the images (20, 21). With small datasets, deep neural networks will inevitably overfit, i.e., perfectly model the training data but lack generalizability and therefore perform poorly in a different validation dataset (21). However, there is substantial difficulty in obtaining a large gestalt dataset when privacy concerns associated with collecting facial photographic data exist, and especially in the emergency setting (22, 23). The use of simulated or synthetic data and augmenting existing data may solve this problem, as previously demonstrated for medical imaging and electronic medical record data (24–27). Moreover, there is vast literature, including recent studies, highlighting several key features of acute illness – including “a tired appearance,” “pale skin and/or lips,” “swollen face,” and “hanging eyelids” – which can accurately be simulated (28–31).

Thus, to get insight into the usability of gestalt data in categorizing sick individuals, we used facial photographs of volunteers simulating these features to represent persons with and without acute illness. We trained a deep learning algorithm on facial photographs of simulated acute illness and a dataset of augmented facial photographs using a style transfer algorithm. Then, a concatenated model with multiple convolutional neural networks was validated on an external dataset of photographs of otherwise healthy volunteers injected with lipopolysaccharide (LPS).

## Methods

### Dataset

**Figure 1:**
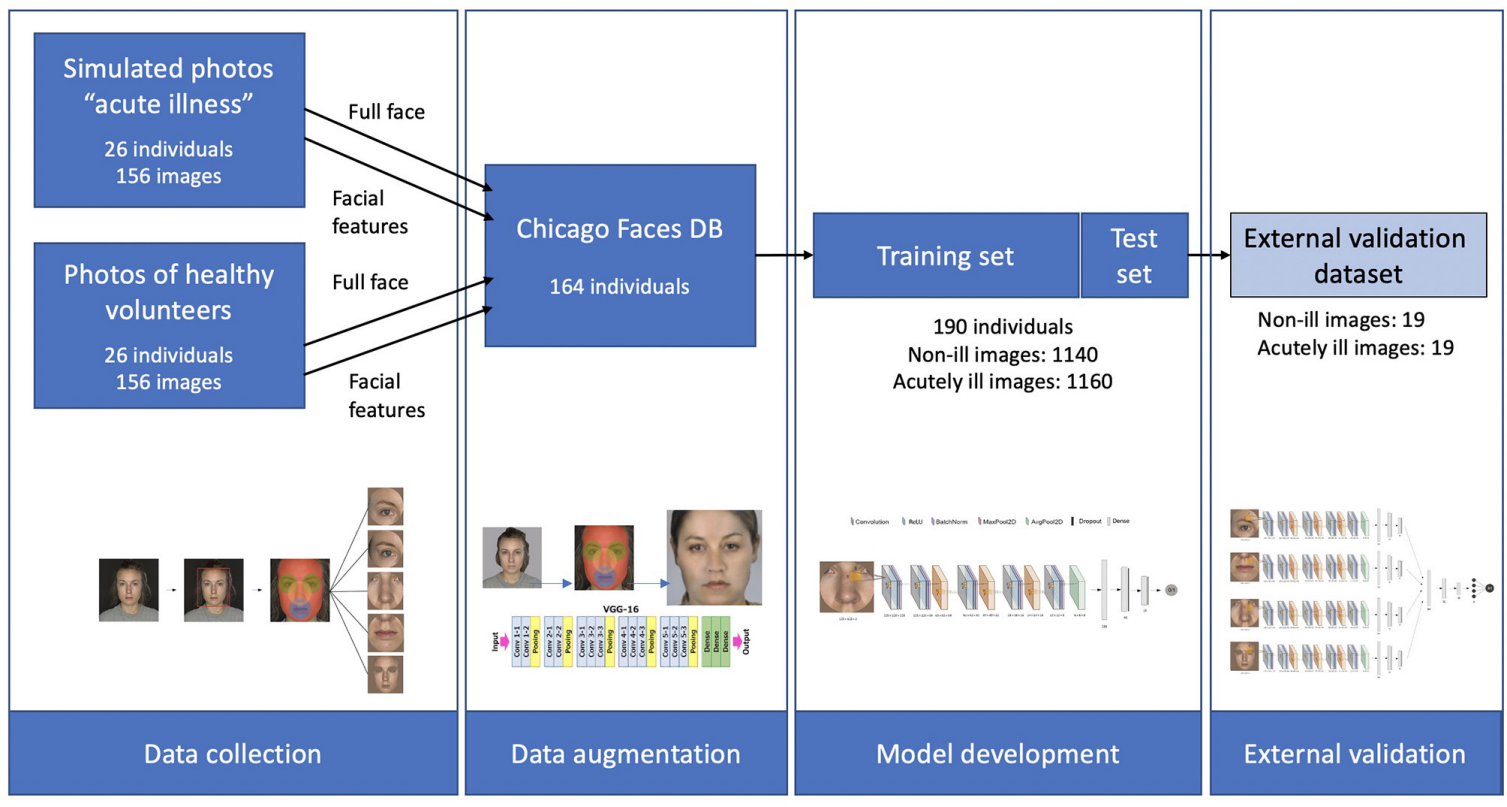
Schematic overview of the three datasets, and of the different steps in the study. Starting with data collection from 26 healthy individuals and simulated acute illness. These features were then extracted and transferred to 164 faces from the Chicago Faces database, resulting in a total of 2,300 images of healthy and acutely ill individuals. After training the deep learning algorithm, validation was carried out on an external dataset of 19 individuals using a stacked CNN combining 4 individual networks.

An overview of the different steps of this study is provided in Figure 1. Three different data sources were used. The training dataset was created through combining two sets of photographs. First, a set of “simulated” sick faces, where the facial features of healthy volunteers had been manipulated using make-up, and second, a set of synthetically generated data resulting from the transfer of these features onto photographs from an open-source faces database (32). The validation dataset used data from a third set of photographs, which consisted of facial photographs from a previous study of individuals before and after they were administered LPS to experimentally induce acute illness (33).

#### Dataset With Simulated Sick Facial Features on Healthy Volunteers

Facial features characteristic of acute illness were simulated using make-up on 26 individuals (11 female). These characteristics of early acute illness included changes in skin color (pallor) due to vasoconstriction, drooping of mouth corners, and eye closure, often due to altered mental status (28–31). In total, seven facial features were simulated: paler skin tone, pale lips, redness around the eyes, sunken eyes, redness around the nasal alae, droopy mouth, and more opaque skin. The standard protocol followed for the make-up application is shown in Supplementary Table 1 and Supplementary Figures 1–3. Two photographs of each participant were selected and included in the study, one without any make-up to represent the “healthy” control state, and another to represent the “acutely ill” state. A standardized environment with a gray background and LED light was used, and photographs were taken with an iPhone 8 camera (4,032 × 3,024 pixels) with standardized settings (ISO 22, RAW, AF, S1/40, MF: 0.9 and AWB in the Halide app). White balance of the complete set of photographs was standardized by a professional photographer using Adobe Photoshop (CC 2019).

#### Data Augmentation to Expand Training Dataset

To expand the dataset, one hundred sixty-four distinct faces from the Chicago Face Database (CFD) were retrieved and taken to represent “non-sick” individuals (32). In addition, photographs mimicking acute illness were generated using the same individual faces from the CFD and a neural algorithm of artistic style transfer. This algorithm transferred the make-up style representing acute illness to healthy individuals from the CFD. A VGG19 deep convolutional network was trained so that it got exposed to each image for 1,500 steps. Male and female participants were separated to ensure appropriate transfer of features and lower artifact creation. The one image per subject visually assessed by two researchers (JCF and AV) to represent the best acute illness was selected.

#### Validation Dataset of Individuals With LPS-Induced Illness

The external validation dataset consisted of the photographs of 22 individuals before (placebo, healthy) and 2 h after being injected with LPS. These individuals were mostly male (9 female) and of a similar age (mean 23.4). Camera resolution settings used were similar to those described before, and an equally standardized procedure was followed using a studio set-up. Additional details of these data are provided elsewhere (33).

### Ethics

The study was exempt from ethical approval from the Medical Ethical Committee of the University Medical Centre Groningen. For the healthy volunteers, consent was obtained from all volunteers, including for the use of certain images for publication. A license for the use of the CFD was obtained by the study's authors (JCF and AV). Lastly, consent for collection and use of the photographs in the validation set was obtained previously, with the original study being approved by the regional ethical review board of Stockholm, Sweden (Registration number 2015/1415-32) and registered in ClinicalTrials.gov (NCT02529592) (33).

### Data Pre-Processing

**Figure 2:**
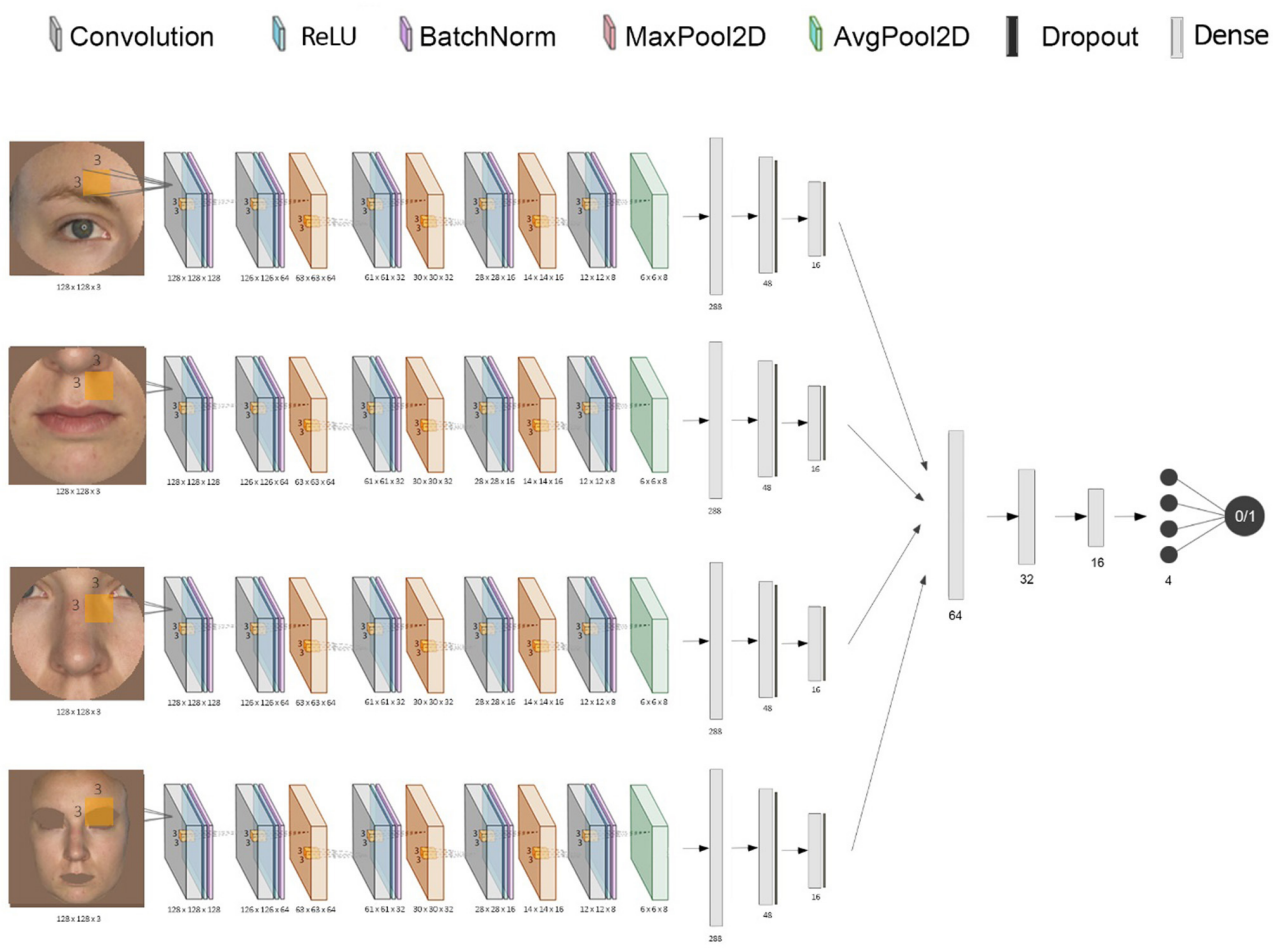
Diagram of the stacked CNN. This shows the combination of each CNN's inputs and outputs into one final binary classification of “acutely ill” or “healthy”.

The simulated photographs and the validation photographs differed in certain aspects. In the simulated data, the features of acute illness were more accentuated than in the LPS group. In addition, the lighting was brighter in the validation data set, with somewhat dimmer light and more pronounced shadows and contrasts in the simulated dataset. To correct for this, all photographs in the simulated set were brightened (gamma = 1.3). All photographs were then resized to 128 × 128 pixels, and the four facial features (eyes, nose, mouth, and skin) were extracted separately using computer vision algorithms, as shown in Figure 2. A Haar cascade facial classifier was used to identify the entire face region in an image (34, 35). The facial landmark detector identified the face features, obtained by training a shape predictor on a labeled dataset (36, 37). The eyes, nose, and lips were extracted by calculating the minimum circle enclosing the 2D set of points representing each feature (given by the facial landmark detector). Finally, the skin area was extracted by removing the eyes and lips regions and everything outside the jaw region. Any other background and hair were removed by thresholding out certain color ranges (between HEX #000000 and #646464; #a0a0a0 and #aaaaaa were selected based on observation). The removed regions were replaced with the dominant color calculated from each face region, ensuring no other noise is passed down through the CNNs.

### Deep Learning Algorithm

A CNN was trained for each facial feature using Keras with a Tensorflow backend. The individual networks input is represented by a 128 by 128 pixels RGB image, which is convolved with a convolution kernel of size (3, 3) after adding padding, using 128 filters. We use a rectified linear unit (ReLU) as an activation function, the output being normalized and scaled through a layer of batch normalization. The subsequent layers progressively down-sample the image data through groups of convolution layers (without padding), batch normalization, and max pooling layers with a pool size of (2, 2). Then, the final down-sampling layer uses an average pooling layer (with the same pooling size) to smooth the resulting filters. Finally, the output is flattened, resulting in a tensor of length 288. This is passed through two other fully-connected layers, each having a drop-out layer. The final layer is fully-connected with the output unit that uses a sigmoidal activation function, which generates an output value between 0 and 1 representing the probability of being classified as “ill.”

To build the stacked ensemble combining all the previously mentioned CNNs, the final layer of all individual networks was removed, and each vector representation of size 16 was concatenated, resulting in a vector of size 64 (Figure 2). The data was then again gradually down-sampled through four fully-connected layers using ReLU (of size 32, 16, 4, and 1, respectively). The final activation function for the output is again the sigmoid function to ensure a value between 0 and 1. Both the CNNs and the stacked network use an Adam optimizer (adaptive moment estimation) with an initial learning rate of 0.001 and values for beta1 = 0.9, beta2 = 0.999, and epsilon = 10^−8^. All models used a binary cross-entropy loss function. In order to minimize overfitting, early stopping and model checkpoints were used to save the model with the best testing F1 score during training.

### Statistical Analysis

**Figure 3:**
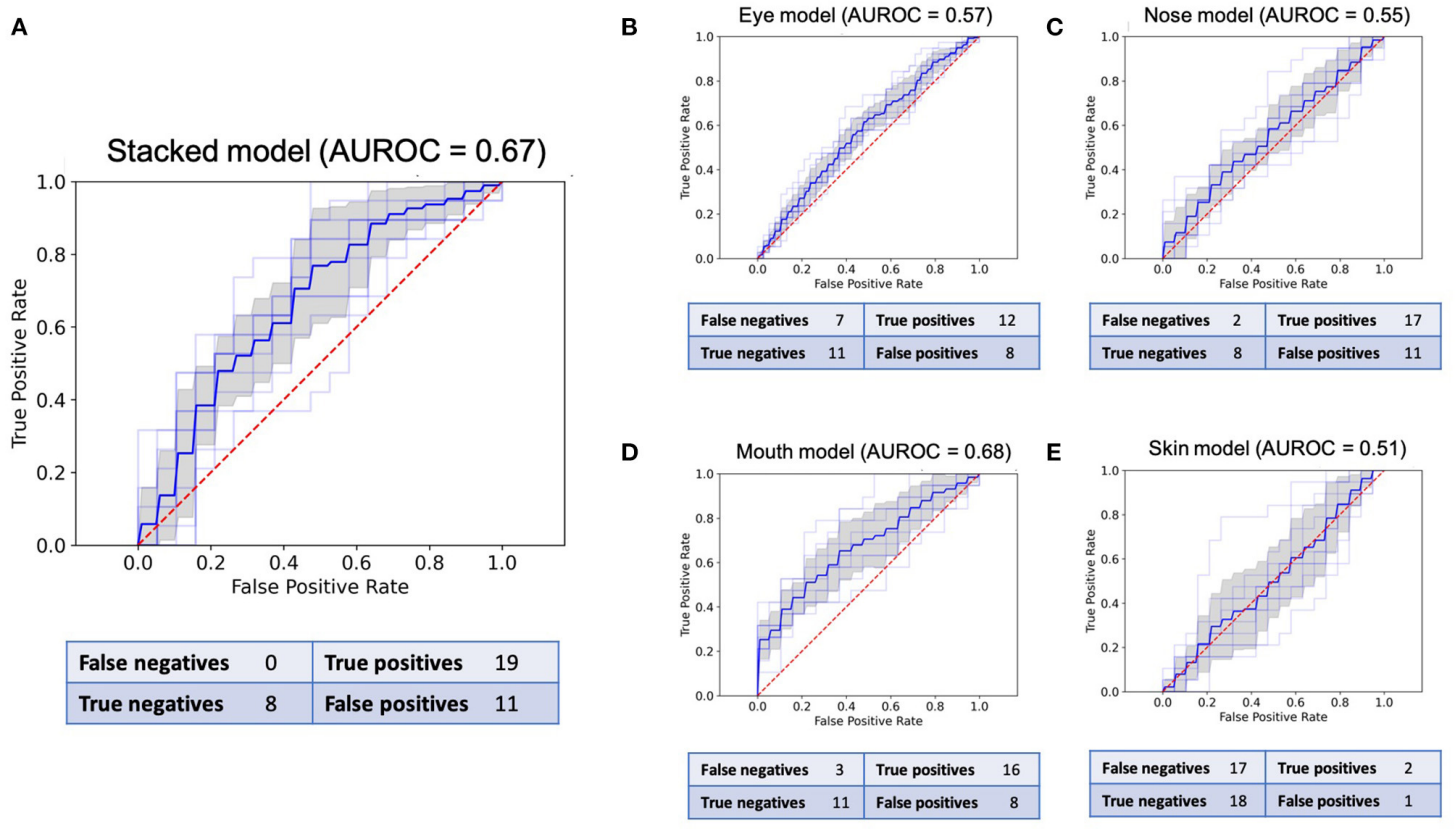
Receiver operating characteristic (ROC) curves and confusion matrices for the final model of all five CNNs in the validation set of 38 images. **(A)**: ROC for the stacked model. **(B)**: ROC for the eyes. **(C)**: ROC for the nose. **(D)**: ROC for the mouth. **(E)**: ROC for the skin.

Each CNN was trained using 10-fold cross-validation. The best model with regard to testing accuracy across all folds was used to make predictions on the validation data. The different CNN models' performance is reported as the respective area under the receiver characteristic operating curve (AUROC), sensitivity, specificity, and negative and positive predictive values on the external validation data (38). Box-and-whisker plots were used to represent the median and interquartile ranges (25–75%) of all model AUROCs. All results are presented with a 95% confidence interval. Confusion matrices aggregating the predictions made by the final models are provided in Figure 3.

## Results

After data augmentation, the training dataset included photographs from 190 distinct individuals, adding up to a total of 1,140 healthy images and 1,160 images representing a state of acute illness for different facial regions, as well as for the complete face.

The sensitivity and specificity reported for each model pertain to the best models in the binary classification task and are based on the confusion matrices presented in Figure 3. The stacked CNN achieved an AUROC in the validation dataset of 0.67 (95% CI 0.61–0.72), with a sensitivity of 100% (82.4–100.0%) and specificity of 42.1% (20.3–66.5%). With regard to the four CNNs trained on individual features, the network with the best performance at distinguishing between healthy and ill individuals was the mouth CNN, with an AUROC of 0.68 (0.62–0.74) and sensitivity of 84.2% (60.4–96.6%) and specificity of 57.9% (33.5–79.8%). All other CNNs achieved AUROCs between 0.51 and 0.57, with sensitivities between 10.5% (1.3–33.1%) and 89.4% (66.9–98.7%), and specificities between 42.1% (20.3–66.5%) and 94.7% (73.9–99.9%). The positive predictive values (PPV) for individual models ranged between 60 and 66.7% for the nose and mouth models, respectively (Table 1). The negative predictive values (NPV) ranged between 51.4% for the skin model and 80% for the nose model. For the stacked model, PPV was 63% (54.1–71.7%) and the NPV was 100%.

**Table 1:** Performance of the best models for each feature and the stacked model on the validation set.

**Trained on CFD augmented with simulated acute illness photographs**					
**Model**	**AUROC**	**Sensitivity (%)**	**Specificity (%)**	**PPV (%)**	**NPV (%)**
Mouth	0.68 (0.62–0.74)	84.2 (60.4–96.6)	57.9 (33.5–79.8)	66.7 (53.3–77.8)	78.6 (54.8–91.7)
Nose	0.55 (0.50–0.60)	89.4 (66.9–98.7)	42.1 (20.3–66.5)	60.7 (50.6– 70.0)	80.0 (49.3–94.3)
Skin	0.51 (0.43–0.59)	10.5 (1.3–33.1)	94.7 (73.9–99.9)	66.7 (16.5–95.3)	51.4 (46.8–56.1)
Eye	0.57 (0.55–0.59)	63.2 (38.4–83.7)	57.9 (33.5–79.8)	60.0 (44.4–73.8)	61.1 (43.8–76.0)
Stacked	0.67 (0.61–0.72)	100 (82.4–100.0)	42.1 (20.3–66.5)	63.3 (54.1–71.7)	100.00

*Values are presented as the area under the curve (AUROC), sensitivity, specificity, and positive and negative predictive values for 50% disease prevalence with 95% confidence intervals*.

*PPV, positive predictive value, NPV, negative predictive value*.

**Figure 4:**
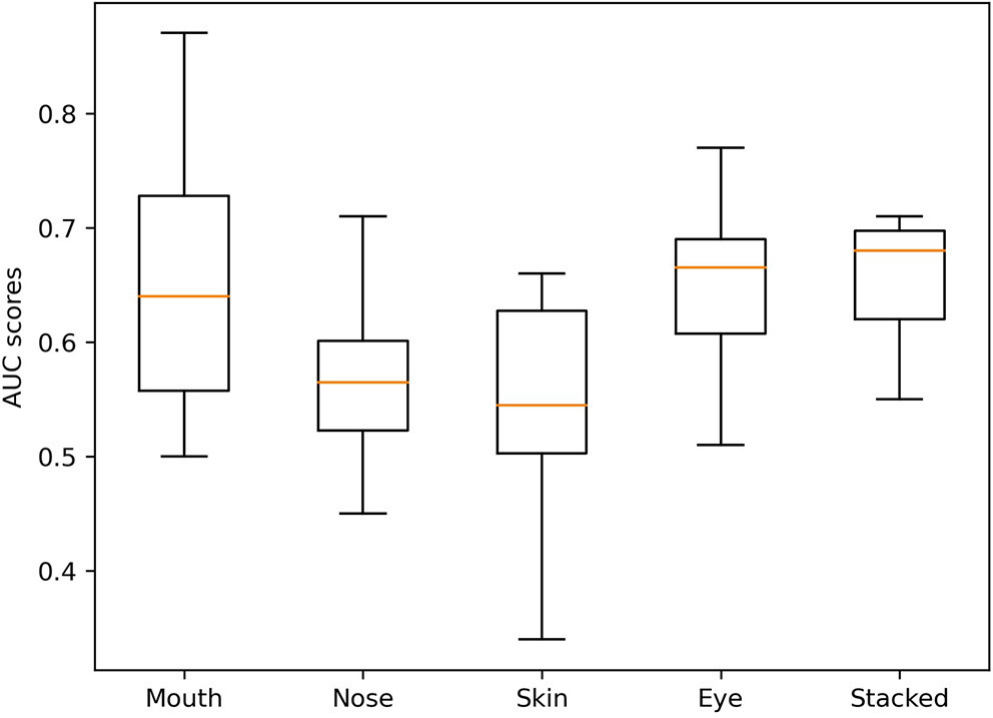
Box-and-whiskers plot of AUC scores of the final models.

The variation in performance of the individual and stacked models in the validation set across the different folds can be seen in Figure 4. Despite the marginally higher AUROC of the best mouth model compared to the stacked model, the stacked model was the most stable across all folds.

## Discussion

In this study, we developed a deep learning algorithm combining multiple convolutional neural networks to distinguish between healthy and acutely ill individuals based on facial feature analysis. We showed that an algorithm trained on augmented facial data of simulated acute illness can successfully generalize predictions on an external dataset of individuals injected with LPS. The final, stacked model combining eyes, mouth, skin, and nose distinguished healthy and ill participants with a sensitivity of 100% (95% CI 82.4–100.0), specificity of 42.1% (20.3–66.5), and AUROC 0.67 (0.61–0.72).

The aim of this study was to investigate how a deep learning algorithm trained on augmented, facial data of simulated acute illness would perform in distinguishing between acutely ill and not ill individuals from an external set of photographs of real individuals with LPS-induced illness. While clinicians or other algorithms' baseline discriminatory ability for acute illness is not established, previous studies on the identification of acute illness based on facial features reported an AUROC of 0.62 (0.60–0.63), with sensitivity and specificity of 52 and 70%, respectively (33). These results were somewhat improved by the stacked model. However, both previous studies on the detection of different acute pathologies by trained physicians, as well as of clinical scores in sepsis detection, have found better results (7, 12, 13). For pneumonia and acute rhinosinusitis, the clinical gestalt achieved AUROCs of between 0.77 and 0.84 (12). Similarly, for acute heart failure, a specific combination of physical cues was converted into a score and achieved AUROCs above 0.90, diagnosing up to 88% of heart failure patients (13). Therefore, we can say this deep learning algorithm trained on simulated “gestalt” data distinguished between photographs of acutely ill and healthy people above chance level, surpassing the performance of non-experts, but fell below the performance of trained clinicians in other studies of different health conditions. This has several potential clinical implications. Firstly, it supports further research on the use of clinical gestalt for detection of acute illness in the ED and ICU, alone or possibly in combination with other clinical parameters. Combining “gestalt” and the modified SIRS score has already been shown to achieve good predictive performance for 24-h mortality in children (39). In adults, the Third International Consensus Definitions for Sepsis and Septic Shock (Sepsis-3) support the idea of combining the adult SIRS criteria and clinical gestalt to screen, triage, and treat patients with infection (15). And secondly, it suggests that adding “gestalt” to other machine learning algorithms for sepsis or septic shock detection may be of value, as these have traditionally focused on vital signs and electronic health record information (40, 41).

In addition, our study reached some technically interesting conclusions related to the feasibility of using synthetic data for deep learning. It is known that the generalizability of deep learning is lower, and the chance of over-fitting conversely higher, in small datasets. This is especially true for imaging data. Therefore, it was an interesting challenge to test whether synthetic data generation and data augmentation could be valid methodologies to address the problem of data availability for certain health conditions in a research setting, be it due to legal-ethical and privacy concerns or to low prevalence of disease (21, 22). We found scarce examples in literature of studies simulating a specific disease-state using techniques such as facial manipulation with moulage or make-up. One other study took photographs of volunteers before and after application of moulage designed to simulate traumatic facial injuries, and found that upon examination of these photographs by a facial analysis software, between 39 and 90% of photographs of injured patients were identified correctly (42). Clearly, synthetic and augmented datasets have the potential to enable researchers to “tailor” data to a specific context, but their generation and use is not without challenges. One immediate challenge is that a definitive measure for the quality of synthetic data is currently lacking (43). Here, we attempted to achieve as great a similarity as possible between training and test data by using a widely validated methodology for feature detection and extraction, and then manually selecting the photographs to be included in the training set (36). Yet, we found that both the deep learning algorithms identified “healthy” individuals with higher accuracy. This was also the case for the non-expert raters in Axelsson et al. 's study, and could be due to an inherently greater degree of similarity between the facial features of healthy individuals than those of the acutely ill ones (33). However, we cannot rule out the possibility that it could also be a reflection of the features of acute illness in the validation dataset being less prominent than in the simulated training data. Because the risk of dissimilarity between training and testing data increases as the size of the dataset increases, and manual verification would not be possible for millions of images, the development of methodologies and standards to measure the quality of synthetic data is necessary before it can be used more widely.

Limitations of this study include the relatively small size of the training dataset, despite the data augmentation process, if compared to established clinical image databases for other diseases (44–46). This prevented us from further tuning the models' hyper-parameters on a holdout subset of the data and may have led to some overfitting. Second, there is a chance the data are inherently biased regarding the illness features and the ethnicity of participants. Despite the standardized, literature-based procedure for acute illness simulation in healthy volunteers, it is possible that individuals whose sick features are naturally more discrete were underrepresented. Equally, both the training and validation datasets included mostly Caucasian individuals, limiting the generalizability of the model to other ethnicities. Further tuning of the model on more ethnically diverse data and testing on a multi-ethnic dataset is warranted (47). Lastly, the potential for implementation of the algorithm can only truly be assessed in a dataset of real ICU or emergency department patients. While LPS produces physical symptoms similar to sepsis and is a well-acknowledged model to study sepsis in humans (48), real patient photographs collected in the ICU or emergency department would bring different challenges than photographs taken in a simulated setting. This could be due to noisy data from different lighting, wires, respirator tubes, and lower standardization of data.

In conclusion, a deep learning algorithm trained on synthetic data representing the clinical gestalt of acute illness was able to distinguish moderately well-between healthy and acutely ill individuals in an external dataset of individuals with LPS-induced acute illness. These results support the value of clinical gestalt as a diagnostic tool for acute illness. Additionally, synthetically generated data seem to be a valid alternative methodology to develop models for health conditions in which large datasets are difficult to obtain.

## Data Availability Statement

The datasets presented in this study can be found in online repositories. The names of the repository/repositories and accession numbers can be found below: https://github.com/J1C4F8/deep_learning_acute_illness.

## Ethics Statement

The studies involving human participants were reviewed and approved by Regional ethical review board of Stockholm, Sweden (Registration number 2015/1415-32). For generation of the training data, no ethical approval from the Medical Ethical Committee of the University Medical Centre Groningen was needed. The patients/participants provided their written informed consent to participate in this study. Written informed consent was obtained from the individuals for the publication of any potentially identifiable images or data included in this article.

## Author Contributions

CF: main contributor to all aspects of the manuscript. AV and MC: artificial intelligence student, significant contributor to the methods and results sections of the manuscript, and designer of figures of software architecture. GY: artificial intelligence student, significant contributor to the methods and results sections of the manuscript. LA, CE, LF, and LC: medical students responsible for data collection for the dataset of simulated features of illness. RH: significant contributor to the methods and discussion sections of the manuscript. IH: co-supervisor in the clinical aspects of the manuscript and original ideation. MW: supervisor of the model development and significant contributor to the methods and discussion aspects of the manuscript. TS and JA: significant contributors to the methods sections and responsible for creating and providing the validation data. AE: main supervisor in the clinical aspects of the manuscript, original ideation, and significant contributions to the introduction and discussion. All authors contributed to the article and approved the submitted version.

## Conflict of Interest

The authors declare that the research was conducted in the absence of any commercial or financial relationships that could be construed as a potential conflict of interest.

## Publisher's Note

All claims expressed in this article are solely those of the authors and do not necessarily represent those of their affiliated organizations, or those of the publisher, the editors and the reviewers. Any product that may be evaluated in this article, or claim that may be made by its manufacturer, is not guaranteed or endorsed by the publisher.
