# Supplementary material for: Deep Learning for Identification of Acute Illness and Facial Cues of Illness
Source: Front Med (Lausanne). 2021 Jul 26;8:661309. doi: 10.3389/fmed.2021.661309 (PMC8350122; doi:10.3389/fmed.2021.661309)

Supplementary Material

**Data collection**

Data was collected in the form of photographs which were taken in March 2020. Subjects were first photographed without makeup in their considered “non-ill state”. Makeup was then applied to simulate acute illness and individuals were photographed again. The makeup and photographing procedures were both taught and advised by professionals. Details about the photography setup and the makeup procedure are given below. During the working process checklists were used to ensure that photographs were taken under the same conditions and that no makeup steps were forgotten.

***Simulated facial cues of illness***

Facial features of acute illness were replicated using makeup on 26 individuals. These features included: paler skin tone, pale lips, redness around the eyes, sunken eyes, redness around the ala of the nose, droopy mouth and less glossy skin. An early change in skin colour (less red, paler) is a reliable indicator of acute illness as well as a more negative expression identified by drooping mouth corners and eyelids. With the aid of a standardized protocol, the seven facial features were replicated (table S1). Despite standardization, adaptations to the facial features of individuals were made in order to make the “ill-state” look as natural as possible

**Table S1.** Photography preparation protocol

| **Step** | **Facial feature** | **Make-up used** | **Scheme** |
| --- | --- | --- | --- |
| **0.** | None | No make-up was used to take the “non-ill” photograph.    Participants had to remove any other make-up. | 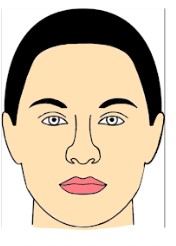 |
| **1.** | Skin tone: | White-toned foundation was used to increase the pallor of the skin. Adjustments were made to adjust this to the natural skin tone by: adding some darker skin tone foundation or color correcting by. mixing green concealer with skin tone concealer and apply on red areas. | 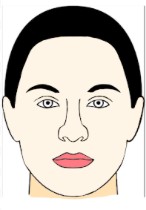 |
| **2.** | Pale lips | A mix of green concealer with skin tone concealer was applied. | 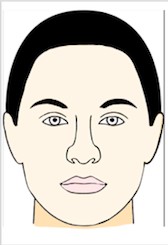 |
| **3.** | Redness around the eyes: | A mix of red, purple and brown lip product was applied. The natural skin lines were respected. More purple was used for lighter skin, and more brown for darker skin. | 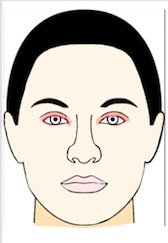 |
| **4.** | Sunken eyes | A brown camouflage cream was used for the natural wrinkles and face lines under the eye, and the lateral corners of the eye with a downward trend of the drawn lines | 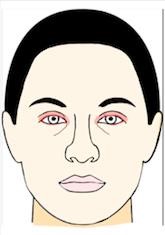 |
| **5.** | Redness around the alae of the nose | A brown camouflage cream was used to colour a triangle form in the corner of the mouth, slightly drawn downwards | 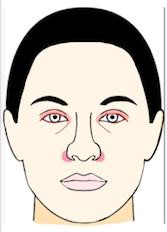 |
| **6.** | Droopy mouth | A brown camouflage cream was used to paint a triangle form in the corner of the mouth, drawn slightly downwards | 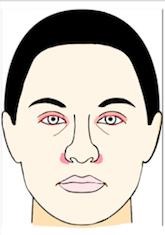 |
| **7.** | Less glossy skin | Light tone (eg. white) powder was applied | 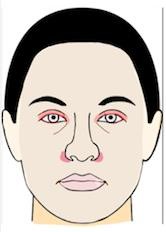 |

***Photographic protocol***

First, a studio-like setup was created using the following material: a gray background (Falcon Eyes Background BCP03, gray, marked as 1 in Figure S1) with the dimensions of 148x200cm; a chair (2 in Figure S1) with a seat height of 45 cm; a color scale (DataColor SpyderCheckr 24: 3 in Figure S1) placed at a height of 105cm; a lighting device (4 in Figure 1) composed of a lamp stand (Caruba LS-1 adjustable lamp stand placed at a height of 78cm) and a LED light (Caruba round Vlogger 45cm LED set economy). The total set up is represented in Figure S1.

**Figure S1.** Schematic representation of the photography set-up
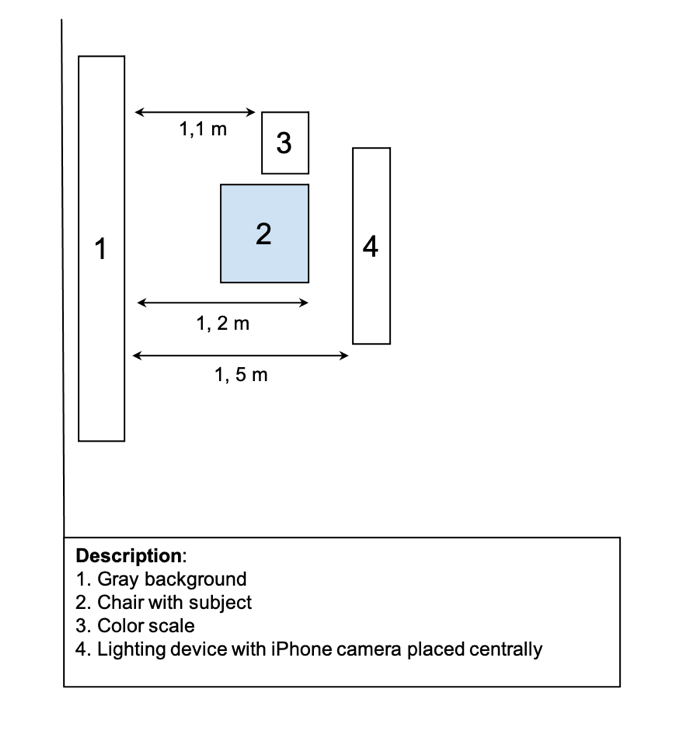


Second, individuals were photographed in the same position (straight back, feet aligned, hands placed on thighs, looking at a fixed point on the lighting device with a natural facial expression for the “non-ill” state and a negative facial expression for the “ill” state. Additionally, a gray T-shirt was worn to reduce discoloration and to optimize light absorption and reflection (Figure S2)*.* Thirdly, the photographs were taken using an iPhone 8, at a camera resolution of 4.032 x 3.024 = 12.192.768 pixels, and settings set to ISO 22, RAW, AF, S1/40, MF: 0,9 and AWB. All photographs were stored in RAW format.

**Figure S2.** Example of a standardized photograph of a male volunteer. Left panel shows the “non-ill” state, and the right panel the “ill” state.


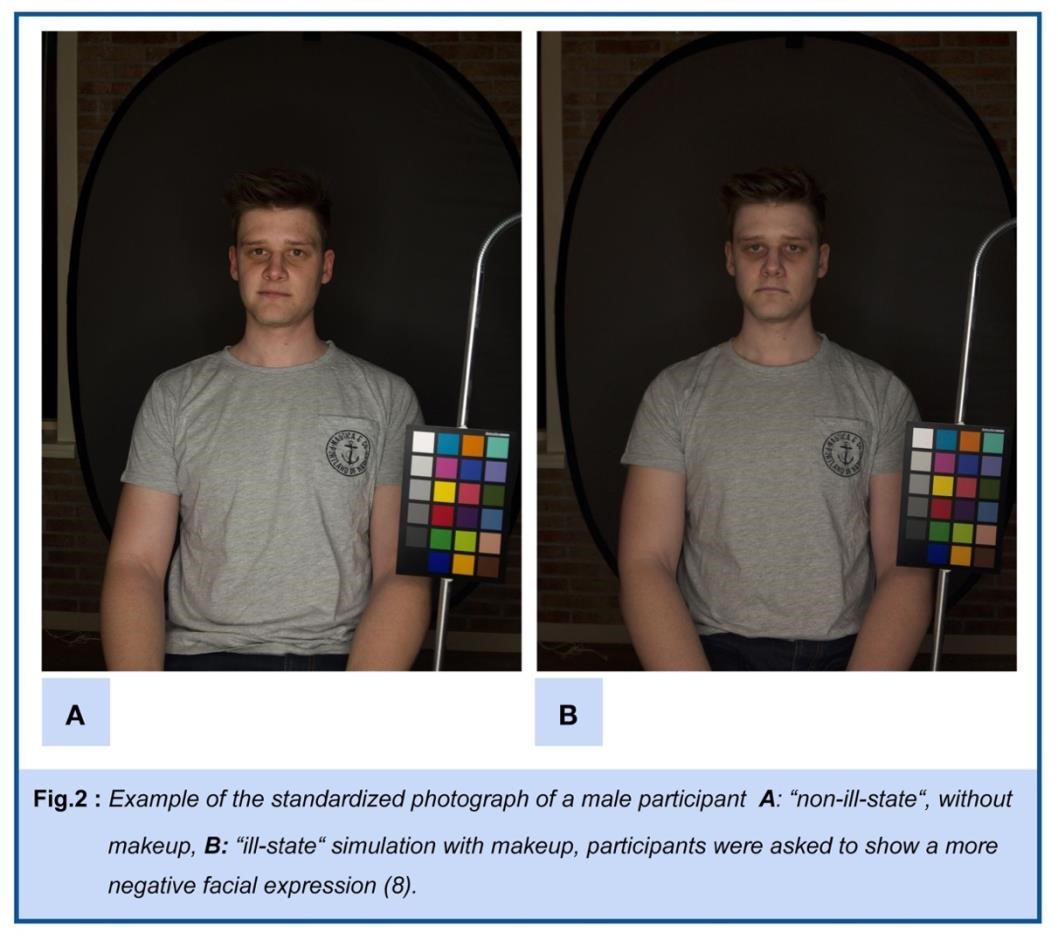


In total 74 photos were included; each individual was photographed with and without make-up. For 22 subjects, a third photograph without makeup and with a different orientation was included to increase the amount of data available to train the algorithm. Afterwards the photographs were labeled “acutely ill” (with makeup) or “well” (no makeup) and divided by gender.

After the photographs were taken, they were prepared with Adobe Photoshop (system-software Mac OX) in order for them to be standardized and adapted to the fit of the photographs from the existing datasets. The RAW file photographs were colour-adjusted (Adobe RGB 1998) based on the colour scale present in the photographs and without compressing them. Interleaved pixel orders were chosen, and the photographs were saved as TIFF files. A white balance correction with help of a white point was made. Consequently, every picture was balanced and had similar tone and colour values. Finally, a rough crop was created (512mmx512mm) from the adjusted photographs and if needed, the rough crop was rotated to be perpendicular (Figure S3).

**Figure S3.** Photograph cropped to 512x512mm and color-balanced. Left panel shows the “non-ill” state, and the right panel the “ill” state.


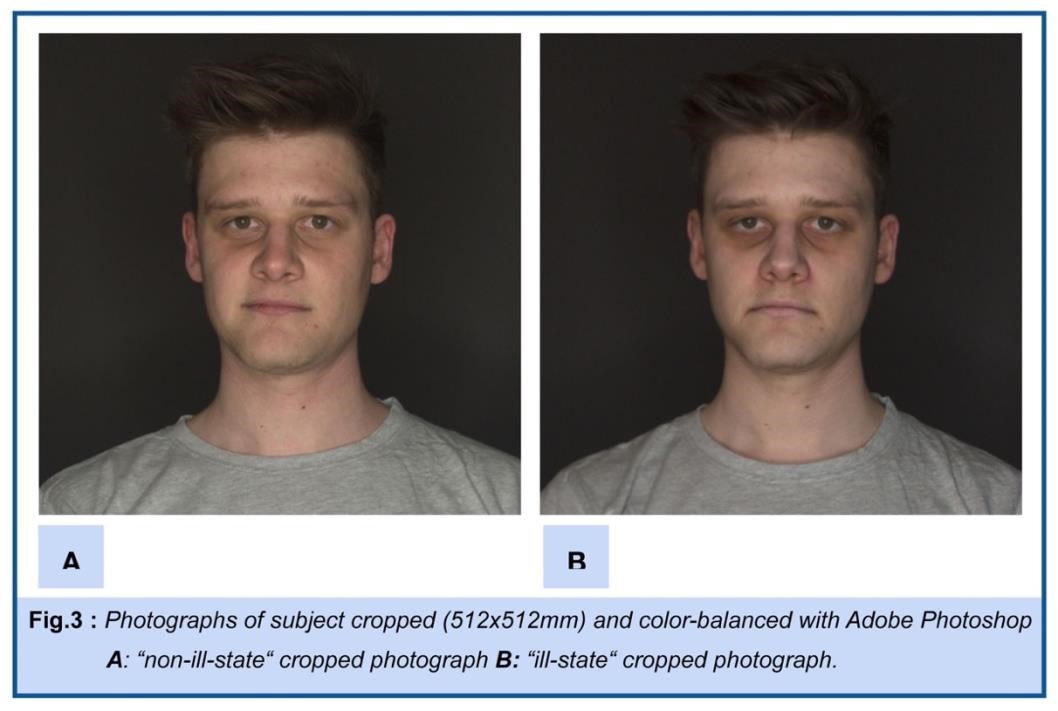

Supplement: Supplementary file 1 [file Data_Sheet_1.docx]
